# Special Issue: “Genes and Human Diseases”

**DOI:** 10.3390/ijms25084455

**Published:** 2024-04-18

**Authors:** Mikhail Churnosov

**Affiliations:** Department of Medical Biological Disciplines, Belgorod State National Research University, 308015 Belgorod, Russia; churnosov@bsu.edu.ru

## 1. Introduction

Studying mechanisms of development and the causes of various human diseases continues to be the focus of attention of various researchers. These studies are of particular importance now and in the near future due to the observed/expected increase in life expectancy of the world’s population and, accordingly, the increase in the number of people suffering from various diseases [1,2]. For example, over the past 25 years (1990–2015), the number of individuals with a systolic blood pressure of 140 mmHg and higher has increased by more than 18% and the associated mortality rate has increased by more than 8% [3]. It has been unequivocally shown that an increase in systolic blood pressure by 10 mmHg causes a significant increase in the risk of cardiovascular diseases (by 45%) and hemorrhagic/ischemic stroke (by 63–66%) [4]. According to the Global Cancer Observatory (International Agency for Research on Cancer of WHO), in the next 20 years (2022–2045), the number of cancer cases among the world’s population will increase by 63% (from 20 million to 32.6 million), and the number of deaths from various malignant neoplasms during this time period will increase by 73% (from 9.74 million to 16.9 million) [5].

Among the large number of diverse factors involved in the development of various diseases, hereditary factors play a significant role in the formation, clinical course, development of complications, and prognosis of disorders in most human diseases, which has been proven, beyond doubt, by numerous genetic studies [6,7]. Twin/family studies have shown that the contribution of heredity to the formation of common human diseases varies widely and can reach 50–80% (for example, for uterine fibroids—55–69% [8,9], polycystic ovaries—79% [10], blood pressure—30–68% [11,12,13,14], endometriosis—47–51% [15,16], etc.). At the same time, the currently known scientific data on candidate genes associated with the risk of developing these diseases (obtained in genome-wide association studies (GWASs) and others) only partially “describe” the genetic determinants of these diseases [17,18,19]. For example, for breast cancer, GWAS loci “describe” only about 44% of the putative genetic determinants of breast cancer (18% of 41%) [20], for endometriosis, the figure is 10% (5.19% of 51%) [21], and for blood pressure the percentage is 31–65% (19.4–21.3% of 30–68% [22], etc.). This indicates the presence of a problem of “unknown [hidden]” heredity and determines the relevance of further genetic studies of human diseases. Additionally, despite the accumulated significant factual material on polymorphisms involved in disease susceptibility (including those obtained in large-scale GWASs; for example, for blood pressure there is information from GWASs on more than 1500 causal loci [7], and more than 220 GWAS significant polymorphic loci are known for their role in breast cancer susceptibility [20,23,24], etc.), these data are often not confirmed in other independent studies and require replicative studies that take into account the ethno-territorial characteristics of the studied population groups, the “specifics” of environmental risk factors, and gene–environmental interactions [25,26]. Furthermore, for a number of diseases, despite their important medical and social significance, data on the genetic nature of these diseases are very few and fragmentary. For example, for endometrial hyperplasia, which is common in perimenopausal women and is a precancerous disorder [27], there are no estimates of the heritability of the disease, GWASs have not been conducted, and data from associative studies are extremely scarce [28]. Additionally, despite the fairly good knowledge of the genes leading to rare/monogenic hereditary diseases, the emergence and development of new genetic technologies for studying genomes (new generation sequencing [29,30], etc.) allow us to obtain new data on the genetic factors (mutations) that cause these diseases, which is of great practical importance [31,32]. So, the above arguments clearly indicate the need to continue active research on the genetic nature of human diseases, which is undoubtedly important not only for understanding the causes/mechanisms of development of these diseases but will also create prerequisites for their introduction into practical medicine.

The Special Issue of “Genes and Human Diseases” presents 13 papers highlighting new data on the role of genetic factors in the formation of various human diseases in order to better understand the genetic and molecular mechanisms of the development of disorders in humans.

## 2. An Overview of Published Articles

Zhegalova’s work (contribution 1) presents experimental data on the spatial organization of chromatin, in which its three-dimensional structure in human chorionic cells is considered), which may underlie transcription regulation disorders in various trisomy (chromosomes 13,16,18). The authors showed changes in the contacts between small/large chromosomes in the presence of trisomy and suggested a lower level of gene transcription (due to reduced chromatin availability) in more compact regions of the genome that occur in the presence of additional chromosomes (these DNA regions are enriched with housekeeping genes).

An experimental study performed by Rao et al. (contribution 2) is devoted to the molecular genetics and phylogenetic analysis of two cases of prostate cancer, which are small cell neuroendocrine carcinoma/ductal adenocarcinoma (case 1) and neuroendocrine carcinoma associated with treatment (case 2). The authors identified both features in genome changes in 1-th (doubling of the entire genome in all samples and focal AR amplification) and 2-nd (loss of 13q/4p/17p, 3p gain) cases of prostate cancer, and based on the results of the phylogenetic analysis, they suggested the presence of common ancestors in small cell neuroendocrine cancer and ductal prostate cancer. The authors note that the doubling of the entire genome in tumor cells may mark an unfavorable prognosis of the disease.

The results of another experimental study, using the whole-exome/transcriptome sequencing method, of the monogenic disease microcephalic osteodysplastic primordial dwarfism type II (an autosomal recessive disease associated with mutations in the *PCNT* gene [21q22]) are presented by Marzano et al. (contribution 3). The authors, after sequencing DNA samples from three patients, found a connection between a number of differentially expressed (downregulation) growth factors, such as IGF1R/IGF2R/RAF1, with the biology of the disease. They also revealed a number of rare disease-associated mutations that were previously uncharacteristic for the disease.

The work of Klyosova et al. is devoted to the search for differentially expressed genes in the widespread and socially significant disease type 2 diabetes mellitus (contribution 4). The authors turned their attention to the genes of glutathione metabolism, the potential connection of which with the disease was shown in previously performed works. The data obtained as a result of a transcriptomic study of diabetic β-cells indicate an important role in the pathophysiology of the disease of the transcriptional activity of genes involved in glutathione metabolism (*PGD*, *ANPEP*, *CTH*, *IDH2*), protein folding (*HSP90AA1*, *RPS19BP1*, *HSP90AB1*, *HSPA1B*, *BAG3*, *HSPA8*, *NUP160*, *NDC1*, *RLN1*), and unfolded protein response (*BID*, *ERP27*, *CREB3L4*).

The study by Bragina et al. (contribution 5) was aimed at identifying apoptosis genes as potential genetic factors underlying the reverse comorbidity between Huntington’s disease and cancer. A full-scale analysis of the gene networks involved in the biology of both Huntington’s disease and carcinogenesis was carried out. The authors established a list of genes that determine the reverse comorbidity of these diseases. The most important of this list of syntropic genes were *PSEN1*, *APOE*, *IL6*, *INS*, *SQSTM1*, *HTT*, *SP1*, *LEP*, *BDNF*, *HSPA4*.

The research by Schmidt et al. (contribution 6) proposed a new alternative method for detecting retinoblastoma-significant mutations in the *RB1* gene (somatic copy number alterations and single nucleotide variations) through method of one-step targeted sequencing. The authors conducted a comparative analysis of the results obtained using their newly proposed method and traditional methods previously used for these purposes (low-pass whole genome sequencing/targeted sequencing and deep whole genome/exome sequencing). They convincingly showed the effectiveness of using their proposed cheaper and less time-consuming method, such as one-step targeted sequencing, in diagnosing the genetic causes of retinoblastoma in children (11/11 patients have different somatic alterations of *RB1* 9 single nucleotide variations, 10 recurrent somatic copy number alterations with 1 *MYCN* gain and 4 focal *RB1* deletions).

In a study by Perdomo-Ramirez et al. (contribution 7), as a result of direct sequencing of exons of two genes such as *SLC2A9* and *SLC22A12* in 21 patients with renal hyperuricemia (a rare hereditary disease associated with impaired urate reabsorption in the proximal renal tubules), mutations that cause the development of the disease in the Spanish population were evaluated. According to the data obtained by the authors, 10 patients had the c.1400C>T [p.T467M] mutation in the *SLC22A12* gene, 10 patients also had a c.374C>T mutation of the *SLC2A9* gene, and 1 patient was diagnosed with a new c.593G>A mutation [p.R198H] in the *SLC2A9* gene. The authors suggest that the appearance of the c.374C>T mutation of the *SLC2A9* gene in the Spanish population is associated with the founder effect.

The article by Ivanova et al. (contribution 8) presents the results of a replicative study of the correlation of polymorphic loci, associated with blood pressure/hypertension in genome-wide association studies, with the risk of hypertension in the European population of Russia. Among the nine studied loci, only one polymorphism (rs1799945 (C/G) *HFE*) made an independent contribution to the development of the disease, whereas the vast majority of the studied SNPs (seven polymorphisms) were associated with the disease only when taking into account interlocus interactions. Using an in silico approach, the authors suggested that the variants associated with the disease may be functionally important for more than 100 genes that play a primary role in immuno-mediated processes, which once again convincingly confirms the essential pathophysiological importance of the immune system in the development of hypertension and dictates the need for therapeutic correction of immune disorders linked with the disease. 

Gilyazova et al. (contribution 9) performed a comparative study of the expression profiles of 22 microRNAs in 21 patients with clear cell renal cell carcinoma in order to search for non-invasive biological markers of the cancer risk and severe course of the disease, followed by replication of the identified patterns in an independent sample of 47 patients. When comparing tumor tissue with normal kidney tissue (renal parenchyma was analyzed), the authors found dysregulation of nine microRNAs, including miRNA-18a, -210, -199b, -642, -483-5p, -200c-455-3p, -582-3p, -487b. In addition, the work revealed specific differences in the microRNA profile between different stages of the tumor (low/high TNM levels) and combinations of microRNAs-483-5p, -210, -200c, -455, as well as between normal kidney tissue and a tumor with low (microRNAs -483-5p, -18a, -642, -210) and high (microRNA-455-3p, -200c, -582-3p) TNM levels. The authors note that the above-mentioned microRNAs may be promising biological markers for use in clinical practice.

In an investigation by Habibi et al. (contribution 10), data on the prevalence of antibiotic-resistant genes in aerosol samples obtained from the residential areas of Kuwait city (indoor/outdoor/interior of hospitals) are presented. The authors revealed the presence of beta-lactam (which was the most predominant), aminoglycoside, fluoroquinolone, macrolide/lincosamide/streptogramin B, tetracycline, and vancomycin-resistance and multidrug-resistance genes in aerosols. The paper shows the peculiarities of the occurrence of the studied genes depending on the season of the year (autumn/winter), whether the environment was indoor/outdoor, and the inhaled/exhaled fractions of hospital aerosols. The authors note that all identified genes have both bacterial (pathogenic) origin and themselves act as carriers of pathogenic forms.

Meng et al.’s review article (contribution 11) examines the role of the tropomyosin family of proteins in carcinogenesis (the review article includes data from 130 previously performed studies over the past 5 years on this topic). Based on a detailed and in-depth analysis of the recent literature, the authors present numerous arguments with detailed pathophysiological justification about the importance of proteins of the tropomyosin family (these proteins are associated with the cytoskeleton/actin) due to their participation in various molecular pathways in the biology of malignant tumors at various stages of cancer formation, including in the processes of cell proliferation/growth/migration and angiogenesis/apoptosis/invasion of tumor cells. The prospects of using proteins of the tropomyosin family as noninvasive biomarkers of various types of malignant tumors (bladder, breast, colon, prostate cancer, neuroblastoma) are indicated.

In another review article by Pandey et al. (contribution 12), based on data from 165 literary sources, the role of the *CLEC16A* gene as a primary regulator of the pathophysiology of neurodegenerative processes (using the example of Parkinson’s disease) and various autoimmune disorders (type 1 diabetes, allergic rhinitis, multiple sclerosis, systemic lupus erythematosus, rheumatoid arthritis, etc.) is considered. The authors convincingly demonstrated the essential role of this gene in the regulation of disease-significant processes such as endocytosis, mitophagy, autophagy, intracellular metabolic processes, immune reactions (HLA-II antigen presentation, etc.), and insulin secretion processes. Various experimental studies (performed on both animals and humans) have shown increased risks of neurodegeneration/autoimmune disorders with insufficient CLEC16A function. The authors point to the prospects of drug induction of phytophagy in individuals with risky variants of the *CLEC16A* gene, in whom the function of CLEC16A is largely lost.

In the final article of the Special Issue, presented by Zhalsanova et al., (contribution 13) in the format of a case report, the results of a genetic study of a one-year-old patient from Tuva (Russia) with multiple fractures (osteogenesis imperfecta type VI; during the first year of life, the patient had 27 different fractures) are considered. As a result of the genetic examination (massively parallel and sanger sequencing were performed), a new mutation c.259_260insCGGCC [p.T87fs] in the homozygous state in the *SERPING1* gene was found in the child, which causes a shift in the current genetic view.

## 3. Conclusions

So, the generalized analysis of the articles published in the Special Issue “Genes and Human Diseases” presented in this paper testifies to the active research activities of various research teams from different countries (China, USA, Russia, UK, Italy, Spain, Kuwait) in this field. Of the 13 papers published in the Special Issue, a significant number of papers (5/13, 38%) are devoted to the study of the genetic mechanisms of carcinogenesis (prostate cancer, clear cell renal cell carcinoma, retinoblastoma, etc.). In three papers, the genetic causes of the development of rare/monogenic forms of hereditary diseases (microcephalic osteodysplastic primordial dwarfism type II, renal hyperuricemia, osteogenesis imperfecta type VI) (23%) are studied, while two papers focus on the complex mechanisms of the genetic determination of socially significant diseases such as type 2 diabetes mellitus and hypertension (15%). In one paper, a new approach to the genetic diagnosis of retinoblastoma in children (one-step targeted sequencing of the *RB1* gene) was proposed. The Special Issue contains original research (the overwhelming number of papers, 10/13. 77%), reviews (2/13, 15%), and a case report (1/13, 8%).

Attention is drawn to the widespread use of modern methods of obtaining genetic data in genetic research (high-throughput chromosome conformation capture technique, whole-exome/transcriptome sequencing, massively parallel/sanger sequencing, etc.), as well as the use of an in silico methodology, which allows for the most reasoned and evidence-based application of data on functional genomics accumulated to date during the implementation of large international research projects, widely presented/available in bioinformatic databases.

At the same time, despite the significant contribution of the works presented in the Special Issue “Genes and Human Diseases” in solving specific scientific medical genetics problems, it is obvious that this represents a small fragment of the “visible part of the iceberg” and it is necessary to continue active research in this area, the results of which will enable a better understanding of the role of genetic factors in the formation of various human diseases and will create the basis for a wider application of genetic knowledge/techniques/technologies in practical medicine. Newly submitted works on this topic will be published in the next Special Issue “Genes and Human Diseases 2.0”.
